# MRI Prognostic Factors in Multiple Sclerosis, Neuromyelitis Optica Spectrum Disorder, and Myelin Oligodendrocyte Antibody Disease

**DOI:** 10.3389/fneur.2021.679881

**Published:** 2021-11-18

**Authors:** Rosa Cortese, Antonio Giorgio, Gianmarco Severa, Nicola De Stefano

**Affiliations:** Department of Medicine, Surgery and Neuroscience, University of Siena, Siena, Italy

**Keywords:** MRI, multiple sclerosis, prognosis, neuromyelitis optica spectrum disorder, myelin oligodendrocyte antibody disease

## Abstract

Several MRI measures have been developed in the last couple of decades, providing a number of imaging biomarkers that can capture the complexity of the pathological processes occurring in multiple sclerosis (MS) brains. Such measures have provided more specific information on the heterogeneous pathologic substrate of MS-related tissue damage, being able to detect, and quantify the evolution of structural changes both within and outside focal lesions. In clinical practise, MRI is increasingly used in the MS field to help to assess patients during follow-up, guide treatment decisions and, importantly, predict the disease course. Moreover, the process of identifying new effective therapies for MS patients has been supported by the use of serial MRI examinations in order to sensitively detect the sub-clinical effects of disease-modifying treatments at an earlier stage than is possible using measures based on clinical disease activity. However, despite this has been largely demonstrated in the relapsing forms of MS, a poor understanding of the underlying pathologic mechanisms leading to either progression or tissue repair in MS as well as the lack of sensitive outcome measures for the progressive phases of the disease and repair therapies makes the development of effective treatments a big challenge. Finally, the role of MRI biomarkers in the monitoring of disease activity and the assessment of treatment response in other inflammatory demyelinating diseases of the central nervous system, such as neuromyelitis optica spectrum disorder (NMOSD) and myelin oligodendrocyte antibody disease (MOGAD) is still marginal, and advanced MRI studies have shown conflicting results. Against this background, this review focused on recently developed MRI measures, which were sensitive to pathological changes, and that could best contribute in the future to provide prognostic information and monitor patients with MS and other inflammatory demyelinating diseases, in particular, NMOSD and MOGAD.

## Introduction

Magnetic resonance imaging has become a key investigation in different scenarios of multiple sclerosis (MS), including diagnosis, monitoring of disease course, and assessment of treatment response (1). Identifying prognostic markers is critical for the management of MS patients at all disease stages. Some valuable MRI measures of focal pathology (e.g., lesion number, volume, and distribution) are already available for predicting MS outcome, and a number of additional measures of neurodegeneration and functional organisation (e.g., brain and cervical cord atrophy, functional MRI abnormalities) had been proposed as reliable prognostic markers in MS, although their use in clinical practise is still challenging (2, 3). Moreover, recent developments in imaging acquisition protocols and post-processing contributed to a better understanding of pathological processes occurring in the central nervous system (CNS) diseases, thus providing new imaging biomarkers which may be useful to rule out other inflammatory demyelinating diseases that can mimic MS.

The aim of this review was to describe the prognostic role of conventional and non-conventional advanced MRI measures, with a particular focus on recently developed techniques, in patients with MS and other inflammatory demyelinating diseases of the CNS. Because of the overlapping clinical and MRI findings with MS, special attention would be devoted to neuromyelitis optica spectrum disorder (NMOSD) and myelin oligodendrocyte glycoprotein (MOG) antibody-associated disease (MOGAD).

Neuromyelitis optica spectrum disorder is an autoimmune astrocytopathy of the CNS with secondary demyelination, which can be associated with a specific auto-antibody against the antigen aquaporin-4 (AQP4) in 50–90% of cases (4). MOGAD is an autoimmune disease of the CNS characterised by the presence of serological antibodies against MOG, a CNS-specific protein located in the outer layers of the myelin sheath (5). MOGAD is a relatively new clinical entity (6) whose clinical phenotype, disease course, and response to treatment are currently being defined. Several clinical and MRI features overlap across the three diseases (6, 7).

In this review, we gave an overview on the prognostic role of MRI, moving from conventional and established MRI techniques to more recently developed measures, which although not yet considered established prognostic markers in MS, could provide additional information about diseases pathogenesis. Finally, we considered novel MRI developments, including the advent of ultra-high field scanners, and suggested future areas of research. For this purpose, this review included scientific literature of the last 10 years from PubMed using the following search terms: multiple sclerosis, neuromyelitis optica spectrum disorder, myelin antibody glycoprotein associated disease, magnetic resonance imaging, prognosis, and pathogenesis.

## Prognostic MRI Measures of Conventional MRI in MS, NMOSD, and MOGAD

**Figure 1:**
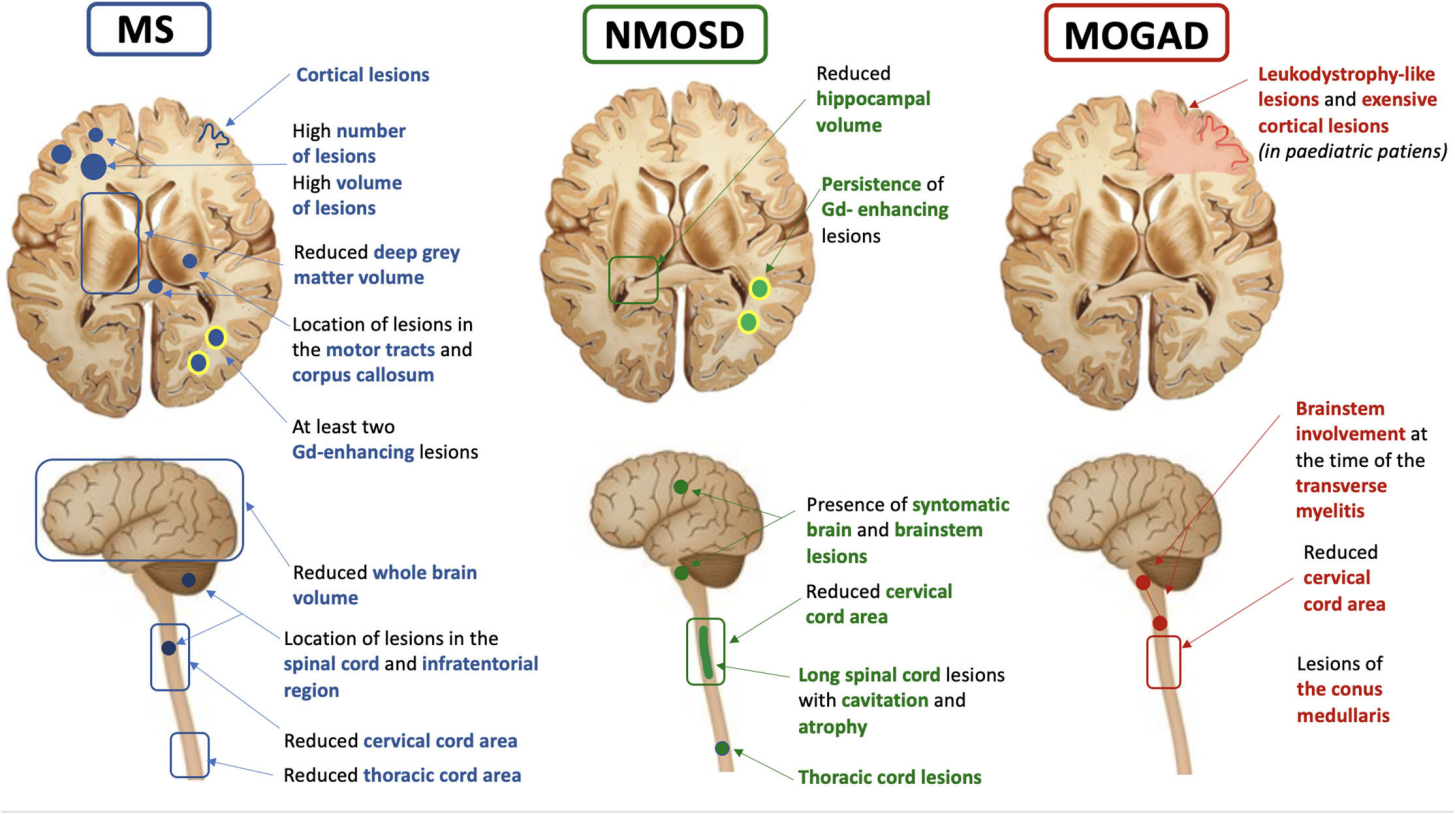
Summary of the most common, clinically available MRI prognostic features in multiple sclerosis (MS), neuromyelitis optica spectrum disorder (NMOSD), and myelin oligodendrocyte antibody disease (MOGAD).

Several MRI measures obtained using conventional MRI sequences have been suggested as prognostic markers in patients with MS. Their ability to predict disease course was consistently found to be higher than that of clinical measures (8). Evidence suggests that white matter lesions (WML), brain, and spinal cord atrophy can help to predict clinical outcomes and monitor treatment response, and several studies over the last years have advanced the field. In this section, we will present the recent impact of these advances on the management of MS, NMOSD, and MOGAD (Table 1, Figure 1).

**Table 1:** Conventional MRI measures with an established prognostic role on clinical disability in multiple sclerosis (MS), neuromyelitis optica spectrum disorder (NMOSD), and myelin oligodendrocyte antibody disease (MOGAD).

**MRI predictors of clinical measures**		**MS**	**NMOSD**	**MOGAD**
White matter lesions	*Number*	*Conversion from CIS to MS:* Higher number at onset	NA	NA
	*Volume*	*Worse EDSS:* Higher volume at onset	NA	NA
	*Location*	*Conversion from CIS to MS:* Lesions located in the motor tracts and corpus callosum at onset *Development of SPMS:* Lesions located in the spinal cord, infratentorial region and deep white matter at onset *Worse EDSS:* Lesions located in the spinal cord at onset	*Long-term disability:* Long, acute spinal cord lesions and symptomatic brain/brainstem lesions *Risk of post-myelitis chronic pain:* Having thoracic (more than cervical) cord lesions	*Poor prognosis:* In paediatric patients, leukodystrophy-like lesions and extensive cortical lesions *Worse outcome:* Brainstem involvement at the time of the transverse myelitis *Long-term sphincteric dysfunction:* Lesions in the conus medullaris
	*Gd-enhancement*	*Development of SPMS and worse EDSS:* At least two Gd-enhancing lesions at onset	*Poor prognosis after an attack:* Persistence of Gd-enhancing lesions	NA
Atrophy	*Whole brain*	*Disability progression and cognitive decline:* Reduced whole brain volume	NA	NA
	*Regional brain*	*Increased risk of disability progression:* Reduced deep grey matter volume	*Cognitive impairment:* Reduced hippocampal volume	NA
	*Cervical cord*	*EDSS progression and gait impairment:* Reduced cervical cord area	*Increased number of myelitis episodes, motor, and sensory disability:* Reduced cervical cord area	*Relapsing course (rather than monophasic):* Reduced cervical cord area

*CIS, clinically isolated syndrome; EDSS, expanded disability status scale; Gd, gadolinium; MRI, magnetic resonance imaging; NA, not available; SPMS, secondary progressive multiple sclerosis*.

### Lesions

White matter lesions count and volume are the most used MRI biomarkers for quantifying the inflammatory activity in MS and represent the primary and secondary efficacy outcomes in many MS clinical trials (9). In patients with the clinically isolated syndrome (CIS), a higher number of T2 lesions at baseline was associated with an increased risk of conversion to clinically-defined MS and T2-lesion volume was able to predict Expanded Disability Status Scale (EDSS) scores at up to 20 years (8, 10). Lesions along specific brain WM tracts involved in motor function and near the corpus callosum were found to be associated with a higher risk of clinical conversion to MS at 1 year (11). In MS patients with at least 1 year of unilateral motor progression and more than five CNS demyelinating lesions, the motor deficit may be attributable to a single critical (i.e., prominent by size and accompanied by focal atrophy) corticospinal tract lesion. This finding supports the role of spinal cord lesions as major contributors to MS clinical motor progression rather than symmetric diffuse brain white matter injury (12).

Recent evidence suggests that lesion topography and gadolinium (Gd)-enhancing lesions are independent predictors of long-term outcomes in patients with CIS. In a cohort of patients with relapse-onset MS, spinal cord lesions, combined with inflammatory activity on MRI at baseline, were found to predict long-term disease outcomes. The presence of spinal cord lesions and at least two Gd-enhancing lesions in CIS patients was associated with a 45% risk of developing secondary progressive (SP) MS after 15 years and with a worse EDSS (13). However, the strongest early predictors, within 5 years of disease onset, of developing secondary progressive multiple sclerosis (SPMS) after 30 years were the presence of infratentorial lesions at baseline and deep WM lesions at 1 year (14).

In MS, especially in the progressive phases, some pre-existing T2 lesions may show chronic activity, which can be associated with clinical progression (15). Histopathologically, these lesions exhibit expansion at the border, revealed by the presence of a rim of iron-containing cells and reflecting tissue loss in the absence of an ongoing acute inflammation (16). A higher number of rim-positive lesions was associated with clinical relapses in relapsing-remitting (RR) MS and patients with at least 4 rim-positive lesions reached motor and cognitive disability at a younger age (17), thus, suggesting that iron rim might represent a risk factor for MS patients.

Recent MRI studies using deformation-based techniques (i.e., Jacobian maps), allowed us to identify in MS those WMLs with a constant and concentric volume increase and defined them as slowly expanding lesions (SELs). SELs represents a promising marker for chronic active lesions, as they can be detected on routinely acquired MRI scans, and may predict clinical progression (18). Chronic lesion activity, as demonstrated by increasing T1-hypointensity within existing lesions, was the strongest predictor of disability progression rather than brain volume loss or the rate of accumulation of new T1 or T2 lesions in a large, prospective imaging study on over 500 patients with primary progressive multiple sclerosis (PPMS) acquired during the ORATORIO trial (18).

In patients with NMOSD, several MRI features, including spinal cord T1-hypointensity, cavitation, or atrophy, were associated with a higher risk of poor recovery, refractory pain, and permanent disability, and lesions in the upper cervical region extending to the brainstem carried a risk of respiratory failure (19). In a recent multicentre study, the presence of longer acute spinal cord lesions and symptomatic brain/brainstem lesions were found as the primary contributors to long-term disability in NMOSD, independently from other factors, such as race and centre (20). Moreover, the presence of thoracic cord lesions in NMOSD patients was associated with a higher risk of post-myelitis chronic pain than the presence of cervical lesions, independently of the number of myelitis relapses, lesion length, and lesion burden (21).

The persistence of Gd-enhancing lesions, due to the breakdown of the blood-brain barrier, turned out to be an important predictor of poor prognosis after an NMOSD attack, a finding which can guide treatment options and suggests an adequate short-term follow-up (22).

In a large French cohort of adult patients with MOGAD, those presenting with an abnormal brain MRI had a higher disability at onset than those with a normal brain MRI (23). In addition, in a longitudinal English cohort of adult patients with MOGAD who experienced at least one transverse myelitis episode, the length of the spinal cord lesions was associated with disease severity at onset while the involvement of the brainstem at the time of the transverse myelitis was predicted a worse outcome, and the presence of conus lesions was associated with long-term sphincteric dysfunction (24). In a recent Spanish study on paediatric MOGAD patients, ADEM-like relapses progressing to leukodystrophy-like features, and extensive cortical encephalitis evolving to atrophy were indicative of poor prognosis.

### Atrophy

In addition to lesions visible on conventional MRI sequences, inflammatory demyelinating diseases may lead to neurodegeneration and consequent brain and spinal cord atrophy, which are more related to disability progression. MRI is also sensitive to the detection of brain tissue loss, and this may be qualitatively assessed in the form of macroscopic brain volume contraction, ventricular enlargement, and widening of the cerebral sulci.

MRI techniques able to assess the whole brain and grey matter (GM) atrophy are now widely accessible using automated analysis methods, and recommendations to improve their measurement and interpretation have been suggested (25–27). Furthermore, new approaches assessing longitudinal changes of spinal cord volume with more precise segmentation-based methods (28, 29) and allowing calculation of spinal cord atrophy from brain MRI volumetric images have been developed, thereby reducing scanning time (30).

Brain atrophy is an important feature of MS pathology, mainly reflecting neuroaxonal loss within lesions and normal-appearing tissue (31). It has been observed since the earliest stages of MS and baseline measurements were found to correlate with long-term disability progression (10, 31). Currently, brain atrophy is the most commonly used imaging biomarker for quantifying neurodegeneration in MS clinical trials, alone or in combination with the absence of new T2 or Gd-enhancing T1 lesions (32). Similarly to whole-brain atrophy, GM atrophy is an important prognostic factor in MS, occurring with a similar pattern across all phenotypes and being faster in deep GM followed by cortical lobes of the brain (33). A reduced deep GM volume at baseline was associated with an increased risk of disability progression over time. While relapse onset MS (CIS, RRMS, and SPMS) firstly developed atrophy in cortical GM (posterior cingulate and precuneus), thalamus atrophy seems to appear early in PPMS (33). The thalamus is a central hub connected with several brain regions and its damage is associated with a variety of clinical manifestations in MS, including fatigue, movement disorders, pain, and cognitive impairment (34, 35). Thalamic volume declines consistently during the disease course and is one of the most important predictors of cognitive impairment in MS (36). The recent evidence of slower thalamic volume loss in several recent randomised, placebo-controlled trials, supports the incorporation of thalamic MRI endpoints in future clinical trials (37). Using source-based morphometry, a recent multicentre MAGNIMS study showed that baseline normalised GM volume and cerebellar GM atrophy independently predicted clinical worsening in all MS phenotypes (38).

In addition to baseline measures, the rate of brain volume loss at both whole and regional levels have been correlated with subsequent disability in MS (39), although these changes should not be confounded by the short-term “pseudoatrophy” that may occur after initiation of some disease-modifying treatments (DMTs) (40). Global brain and GM atrophy changes were mild in a unique group of patients with long-standing (i.e., at least 30 years) disease and no or minimal disability, thus supporting the relevant role of GM atrophy in characterising MS patients who may have favourable long-term disease evolution (41).

The so-called “brain-predicted age” (brain-PAD) paradigm has been proposed as a potential prognostic biomarker in MS, to capture the earliest progressive processes from MRI data (T1-weighted images) using machine learning analysis. Indeed, brain-PAD was independently associated with higher disability, younger age at diagnosis, and longer disease duration, irrespective of disease phenotype (42).

In recent years, a number of studies have proposed spinal cord atrophy as a strong predictor of clinical outcomes in MS. Cervical cord area and volume are differently affected in MS phenotypes, with the greatest changes in patients with primary progressive (PP) MS and higher atrophy rates than those detected in the brain (43). In PPMS, for each 1% annual increase of atrophy, it was found a 28% risk of EDSS progression, independently of brain volume loss (43). Some studies highlighted in MS the presence of damage to specific locations, such as cervical cord GM (44). In RRMS, the cross-sectional area of the spinal cord GM was a strong predictor of EDSS score, and a cut-off value of 11.1 mm^2^ was found to be able to differentiate patients with progressive MS (below the threshold) from those with relapsing MS (above the threshold) (45). The inclusion of thoracic cord measurements improved the correlation between cord atrophy and most clinical progression measures and allowed for better subgrouping of spinal cord phenotypes (46).

Differences in atrophy patterns seem to exist between MS and NMOSD. Indeed, while MS patients showed more whole brain and thalamic atrophy than NMOSD and healthy controls, NMOSD patients showed more spinal cord atrophy and milder brain atrophy, especially in the WM, than healthy controls, thus supporting the presence of different underlying pathogenic mechanisms (47). Therefore, spinal cord atrophy has become a hallmark of NMOSD, being associated with the number of myelitis episodes and lesion length. Spinal cord atrophy on MRI was topographically associated with lesions and correlated with motor and sensory disability (48) unlike MS, where atrophy is only partially related to the presence of focal lesions (49). In NMOSD, progression of spinal cord atrophy was associated with disability worsening and for this reason, it was suggested as a potential biomarker for clinical trials (47).

Different patterns of brainstem atrophy were also observed in the two diseases, with the midbrain being most severely affected followed by pons in MS whereas only the medulla oblongata was affected in NMOSD (50). GM atrophy is another disease-related feature that has been assessed in NMOSD. Indeed, Calabrese and colleagues found in NMOSD patients some mild thinning in the post-, precentral gyri, and calcarine sulcus, which was nonetheless significantly larger than in MS (51). Duan et al. also found GM volume reduction in several regions of the frontotemporal cortex, right inferior lobules, and right insula but it was only significant without correction for multiple comparisons (52). Similarly, a study using voxel-based morphometry found a significant reduction of GM volume in NMOSD patients when compared with healthy subjects, especially in the visual and motor areas as well as in the regions involved in language and executive functions (53). In a study aimed to assess clinical and structural MRI markers for predicting cognitive impairment, hippocampal volume resulted from the main MRI predictor of cognition in NMOSD (54).

Recently, distinct structural brain alterations were identified in MOGAD. Indeed, they included atrophy in the fronto-orbital cortex, temporal gyrus, and deep GM, with hippocampal atrophy correlating with clinical and cognitive disability (55). A significant volume loss in the deep GM structures in MOGAD was found, which correlated with persistent brain lesions (56). When considering the three diseases together, the greatest level of atrophy in the cervical and thoracic cord, particularly in the cord GM, was found in patients with AQP4-NMOSD. In MOGAD patients, volumetric cord measures were lower in those with a relapsing course, even in the case of relapses not involving the spinal cord (57).

## Prognostic MRI Measures of Advanced MRI Techniques in MS, NMOSD, and MOGAD

Important pathological abnormalities occur in MS beyond MRI-visible focal lesions and atrophy. These include neuroaxonal dysfunction and loss, microglial activation, and astrogliosis (58). A number of non-conventional MRI measures have shown sensitivity toward such pathological findings and might provide new prognostic biomarkers in inflammatory demyelinating diseases. Although currently, these novel MRI measures are not well-established as prognostic markers in MS, the application of advanced structural, functional and metabolic imaging techniques to such diseases helped to yield important pathogenetic insights. This is particularly true in NMOSD and MOGAD where, although longitudinal studies of advanced MRI are lacking, some measures have shown clinical relevance in cross-sectional studies. Therefore, in this section, we will report recently developed and promising MRI techniques, which have helped to detect different components of disease pathogenesis *in vivo*, even when their prognostic role was not yet demonstrated (Table 2).

**Table 2:** Advanced MRI measures reflecting different pathogenic mechanisms with a potential prognostic role on clinical disability in multiple sclerosis (MS), neuromyelitis optica spectrum disorder (NMOSD), and myelin oligodendrocyte antibody disease (MOGAD).

**Advanced MRI techniques**		**MS**	NMOSD^*^	MOGAD^*^
Structural	*DIR/PSIR*	At onset, cortical lesions predict aggressive disease course and rapid conversion to SPMS	Cortical lesions are typically absent	NA
	*Diffusion imaging*	Altered DTI measures at onset in brain, spinal cord and optic nerve predict worse EDSS, worse recovery from relapses and decline in vision. Altered QSI measures in early PPMS predict worse postural stability, greater vibration dysfunction and spasticity	Altered DTI measures at cord level involved in the acute attack and WM disruption in optic radiation and corona radiata	White matter disruption in optic radiation and anterior/posterior corona radiata. No alteration in DTI measures detected at cord level
	*Myelin-sensitive imaging*	Changes in MTR values may reflect demyelination or remyelination, correlating with clinical worsening or improvement, respectively. MTR and T1/T2-ratio can be influenced by treatments.	Reduced MTR in the cervical cord (lesional and non-lesional).	NA
	*Connectomics*	Structural network disruption in CIS is associated with early conversion to MS.	NA	NA
Functional and Metabolic	*Functional MRI*	Functional impairment in the thalamus and between thalamus and cortex are associated with cognitive impairment.	Functional impairment and adaption in the thalamus, caudate nucleus and some frontal brain regions.	Functional impairment and adaption in the posterior cerebellar lobe and in the temporal gyrus, respectively.
	*PET*	Using different radiotracers, TSPO-PET, increased uptake inside and around white matter lesions is associated with disability worsening.	NA	NA

* *MRI measures showing relevance in cross-sectional studies are reported*.

*CIS, clinically isolated syndrome; DIR, double inversion recovery; DTI, diffusion tensor imaging; MTR, magnetisation transfer ratio; NA, not available; PPMS, primary progressive multiple sclerosis; PSIR, phase-sensitive inversion recovery; QSI, Q-space imaging; SPMS, secondary progressive multiple sclerosis; TSPO-PET, positron emission tomography with radiotracers targeting translocator protein*.

### Advanced MRI Techniques Assessing Structural Changes

Over the past decade, the relevance of cortical GM pathology in MS has become increasingly recognised. Cortical lesions (CLs) detected at “clinical” field strengths using specific MRI sequences (i.e., double inversion recovery [DIR] or phase-sensitive inversion recovery [PSIR]) is an early and frequent phenomenon in MS and correlate with a disability, cognitive impairment, and a higher risk of transition to the secondary progressive phase (59). Patients with at least seven CLs at disease onset showed an aggressive disease course and a rapid conversion to SPMS while none of the patients without CLs at onset entered the SP phase (59). However, the current detection of CLs *in vivo* is incomplete, capturing only 10–15% of cortical demyelination, even at ultra-high field (UHF) MRI (60). An important aspect to be considered is that most of the lesions detected on MRI are cortico-subcortical or intra-cortical while the most abundant subpial lesions remain largely unrecognised. Cortical lesions are typically absent in AQP4-NMOSD (51), while a cortical involvement in MOGAD has been initially described in the context of ADEM (61). Then, a newly recognised phenotype of benign, unilateral cerebral cortical encephalitis has been recognised (62).

Despite the exact underlying mechanisms are not completely understood, studies on the post-mortem MS brain tissue support the idea that meningeal inflammation and the consequent cortical microglia activation lead to progressive cortical demyelination and neurodegeneration (63, 64). Recently, using high-resolution 3D post-Gd T2-FLAIR images, it was possible to non-invasively detect cortically-based leptomeningeal infiltrates (LME), which in MS were associated with older age, longer disease duration, higher EDSS, greater whole brain, and cortical atrophy (65). However, meningeal enhancement cannot be considered a specific feature of MS since it can also be found in inflammatory, immune-mediated, and infectious vasculitis.

A growing body of work has investigated the role of iron in MS pathogenesis and evolution. The paramagnetic rim is due to the presence of iron-laden activated microglia at the lesion edge and can be easily seen on 7T phase images. Using high-resolution susceptibility-based imaging, nearly all 7T paramagnetic rims can also be seen at 3T, suggesting the possibility of implementing them as an outcome measure in MS MRI-based clinical trials (66).

Moreover, quantitative MRI techniques allow iron quantification *in vivo*. Using quantitative susceptibility mapping (QSM) deep grey matter iron was found to be associated with the secondary progressive course in (MS), independently of tissue atrophy (67). In particular, an increased thalamic iron content, as detected at 7T MRI, was found in patients with MS when compared with healthy controls, which was associated with higher disability scores (68). Several longitudinal studies using advanced MRI techniques showed diffuse injury in the MS normal-appearing brain and spinal cord from the symptoms onset to the progressive stages of the disease (69, 70). Diffusion imaging techniques have been extensively used in MS to assess neuroaxonal integrity in both lesional and non-lesional tissue and along specific WM tracts (71, 72). These techniques include diffusion tensor imaging (DTI) and new models of diffusion. DTI metrics can predict disability progression (73) and cognitive decline (74). In relapsing-onset MS, altered DTI measures in the normal-appearing white matter (NAWM) of the callosum were able to predict disability progression over 4 years (73). Moreover, in patients with CIS and early MS, reduction in fractional anisotropy (FA), a marker of microstructural integrity, in the cerebellum and the cerebral peduncles correlated with EDSS at 2 years (75). Thalamic DTI changes were found to be predictors of disability score deterioration in MS patients followed up for 15 months (76), and they can be used to predict cognitive impairment at 5-years of follow-up (74). In the case of acute spinal cord relapses, higher FA and lower diffusivity in the direction perpendicular to the main fibre direction [i.e., radial diffusivity (RD)] predicted better functional recovery from the relapse (77).

Moreover, increased optic nerve RD was associated with a decline in vision after optic neuritis (ON) and correlated with clinical disability in patients with spinal cord lesions (78).

New diffusion imaging models have been developed in the last years and showed interesting results in MS. An increase over time in the RD of the cervical cord of early PPMS was detected using Q-space imaging (QSI), an advanced model-free diffusion imaging technique with higher sensitivity for neuroaxonal alterations than standard DTI. In the same study, higher RD of the cervical cord predicted worse disability at 3 years, thus suggesting ongoing neurodegeneration in the cord independently of lesions (79). Neurite orientation dispersion and density imaging (NODDI) is a multi-compartment model that enables the estimation of more specific indices, such as neurite density, orientation, and a free-water [cerebrospinal fluid (CSF)-like] component (80). NODDI has shown in MS higher specificity and sensitivity to neurodegeneration compared with traditional DTI measurements. A recent study showed that patients with RRMS had lower neurite density index (NDI), suggestive of neurodegeneration, in the brain NAWM and spinal cord WM than healthy controls. In patients, a lower NDI in the spinal cord WM was associated with higher disability (81). If NODDI can be used to predict disease outcome is still unknown, as no longitudinal studies have been performed thus far.

Myelin-sensitive MRI measures are crucial to investigate MS pathology. In a combined MRI-pathology study, magnetisation transfer ratio (MTR) showed a strong association with myelin content, especially in WM and cortical GM lesions (82). In MS acute lesions, an initial decrease was followed by an increase in MTR, thus reflecting a demyelination process followed by partial remyelination. These changes are correlated with clinical improvement and can be influenced by specific therapies (83, 84).

MTR values in NAWM and GM of MS showed a gradient of abnormalities depending on the distance from the surface of the brain (85). These gradients occur early, worsen with time (86), and may improve after immunotherapy (87), suggesting a remyelination ability of some treatments. However, although sensitive to myelin, magnetisation transfer imaging can be influenced by axonal density and oedema, which may reduce its specificity (88).

Recently, the ratio between conventional T1-weighted (T1w) and T2-weighted (T2w) sequences (i.e., T1/T2-ratio) has been proposed as a reliable measure to evaluate myelin integrity as well as dendrite density (89), which would be easily implementable in clinical practise. A significant T1/T2-ratio increase in NAWM and GM has been demonstrated in RRMS patients during the first 2 years of treatment with disease-modifying drugs (90). Other techniques, such as myelin water fraction and T2 relaxometry in the brain and cervical spinal cord correlated in MS with a physical disability at follow-up (91–93) but their role as MS prognostic measures needs to be further clarified.

In the last decade, approaches of network-based connectomics MRI have been developed to explore the relationships between changes in functional or structural networks and clinical measures (94). For example, in a cross-sectional study, measures of structural network disruption explained scores of EDSS and symbol digit modality test (SDMT), a proxy for information processing speed, above measures of tissue atrophy and WM lesions in different MS clinical phenotypes (95). A longitudinal assessment of specific brain networks (e.g., structural covariance networks) further confirmed their relevance in MS, as their alterations in CIS were associated with early conversion to MS (96).

The application of advanced imaging in NMOSD and MOGAD is emerging, but with many controversies and lack of studies assessing changes in these new measures over time, therefore limiting clinical application in the near future.

Several studies have investigated the presence of diffuse and “occult” damage within the NAWM of patients with NMOSD by applying different MRI techniques. Proton MR spectroscopic imaging (MRSI) showed normal N-acetylaspartic acid (NAA), creatine and choline levels within the NAWM, arguing against occult neuroaxonal damage, inflammation, and gliosis (97). By contrast, a recent study using magnetisation transfer imaging showed in NMOSD an abnormal NAWM, with decreased myelin signal than healthy controls (98).

DTI, which is sensitive to microstructural alterations, has been performed in NMOSD and showed contrasting data. On the one hand, no DTI abnormalities were reported in different brain regions, except for the visual and motor WM pathways where a selective trans-synaptic axonal degeneration may occur secondary to destructive lesions in the optic nerves and spinal cord, respectively (99, 100). On the other hand, other studies have reported a decrease in FA within the NAWM of patients with NMOSD (52, 101). Such DTI abnormalities were, however, rather mild and not as severe as in patients with MS. Specifically, axonal damage and diffusion abnormalities along WM association fibres were more severe in patients with MS than in those with NMOSD (102). Using a multiparametric approach, diffuse WM damage throughout brain concentric bands was demonstrated in NMOSD, as reflected by reduced T1/T2 ratio and increased mean diffusivity, indicating astrocyte damage (48).

Some imaging and histopathological studies in NMOSD showed abnormalities and neuronal loss in cortical GM (103). Less is known about deep GM changes in NMOSD, particularly the involvement of the thalamus is controversial. An attack-related volume reduction of specific thalamic nuclei was demonstrated in NMOSD patients with and without a history of ON, which correlated with the number of clinical episodes, retinal damage, and visual function, thus indicating an anterograde degeneration in the afferent visual pathway (104).

In a MOGAD patient, longitudinal clinical and MRI follow-up showed progressive neurologic deterioration without any relapse associated with progressive WM changes, thus suggesting a possible tissue loss over time. Using diffusion imaging, WM disruption in the optic radiation and corona radiata was identified in both MOGAD and AQP4-Ab positive NMOSD, although milder in the former condition (55).

Results from studies applying advanced MRI techniques to the spinal cord in NMOSD are also controversial. When using DTI analysis for the assessment of cervical spinal cord damage in NMOSD, abnormal DTI-derived metrics (especially FA) in the cervical cord of patients with NMOSD (even in the absence of hyperintensities on T2-weighted imaging) were found (105). Moreover, Klawiter et al. demonstrated higher RD within damaged (as assessed on T2-weighted imaging) WM tracts in NMOSD vs. MS, consistent with the more destructive nature of the former condition (106).

Patients with AQP4-antibody disease showed a significant reduction in the cervical cord MTR, FA, and increased mean diffusivity, and the damage was localised to areas of the cord involved in the acute attack. By contrast, MOGAD patients did not show significant differences compared with healthy subjects in any MRI modality (57).

### Advanced MRI Techniques Assessing Functional Changes

Functional MRI is a powerful tool for studying cortical functional reorganisation and brain plasticity. Studies have assessed functional connectivity (FC) abnormalities within the various brain networks in patients with MS, with the aim of identifying trajectories of changes over the disease course and thus increasing the understanding of MS pathology (107). Resting-state functional MRI (fMRI) has identified a number of FC alterations in patients with MS (108). A decreased FC between thalamus and cortical regions and an increased intra- and inter-thalamic FC were demonstrated as the substrate for early cognitive impairment in patients with MS, independently from thalamic volume loss (54). Specific patterns of increased static thalamic connectivity with the sensorimotor network have been recently identified, which were related to disability in MS (109). In patients with paediatric-onset MS with no or minimal disability, FC was reduced in selective brain networks, probably reflecting the exhaustion over time of functional reserve (110).

Cognitive impairment has been demonstrated in NMOSD patients, as a result of functional alterations within specific neuronal circuits (47). Preliminary studies in NMOSD patients demonstrated a reduced FC in the default mode network (DMN) as well as an increased FC in the thalamus, caudate nucleus, and some frontal regions (47). Recently, the cortical functional reorganisation was shown in NMOSD patients at the level of cognitive networks with an overall adaptive role. Exhaustion of compensatory mechanisms is heralded by an FC reduction in the left frontoparietal working memory network (111).

Severe functional impairment in the visual areas and increased FC in the temporal gyrus were also detected in MOGAD (55), which indicates the presence of functional plasticity trying to compensate for the structural damage, as previously demonstrated in MS and NMOSD (112).

### Optical Coherence Tomography

Neurodegenerative changes along the visual pathway are common in both MS (113) and NMOSD (114). Optical coherence tomography (OCT) is an emerging imaging technique that enables the measurement of the neural retina, whose layer thinning reflects an axonal loss.

Cross-sectional studies of OCT have shown that peripapillary global retinal nerve fibre layer (RNFL) and inner retinal layer thicknesses are reduced in MS and correlate with clinical disability and MRI-derived brain volume measures (115). These findings have been confirmed by longitudinal studies showing that RNFL is predictive of a clinical outcome (poor visual recovery) (116) and they also correlate with imaging measures of MS disease activity and severity (117). In addition, OCT also allows measurement of the ganglion cell and inner plexiform layer (GCIPL) complex, whose atrophy appears to mirror MRI-derived whole-brain atrophy measures, particularly GM atrophy, especially in progressive MS (118).

OCT has been extensively used in MS and NMOSD to detect levels of axonal damage after an episode of ON as well as to help to differentiate the two diseases (119). Retinal axonal loss in NMOSD is more severe than in MS and is most commonly related to ON attacks. Although signs of subclinical axonal loss exist in the non-affected eyes of patients with NMOSD, a clear neurodegeneration pattern has been detected in non-ON eyes in MS patients, as a result of primary retinal neurodegeneration or retrograde trans-neuronal degeneration due to lesions along with the optic radiations. The inter-eye peripheral RNFL difference between eyes with or without ON may be useful in differentiating NMOSD from MS (120). In MOGAD, subclinical ON may occur and this may be associated with a significant reduction in the RNFL thickness. Despite equally significant damage to the optic nerve, patients with anti-MOG antibodies have relatively preserved low contrast visual acuity (121).

### Positron Emission Tomography

Other neuroimaging modalities such as positron emission tomography (PET) have the potential to improve our understanding of the mechanisms of progression in MS, thus providing important prognostic information. PET is a non-invasive molecular imaging technique enabling the *in-vivo* detection of the molecular processes involved in neuroinflammation and neurodegeneration (122). In a 4-year follow-up study, increased radioligand uptake in the perilesional NAWM predicted disability progression independent of relapse activity in MS, using mitochondrial 18-kDa translocator protein (TSPO)-PET, a marker of inflammation linked to microglial and macrophage activation in neurodegenerative and neuroinflammatory diseases such as MS (123). Using a new generation TSPO tracer (F-DPA-714 translocator protein), a novel approach to generate individual maps of WM innate immune cell activation was developed. Strong activation of the innate immune system inside WML and in the NAWM correlated with a more severe trajectory of disability worsening in all MS phenotypes (124). However, to date, the use of different radiotracers, varying patient populations, local scanning protocols, and various analysis methods, led to the heterogeneity of PET research, which currently limits the comparison across different studies and reproducibility. Addressing these issues would enable a multicenter approach and help progress PET imaging from the field of research to a clinically relevant imaging biomarker in MS.

## Future Perspectives

### Ultra-High Field MRI

More recently, UHF MRI (i.e., seven Tesla and above) has gained more interest in clinical imaging. Indeed, a number of studies have shown the benefits from the application of this powerful tool not only for research purposes but also in clinical settings to facilitate a correct diagnosis, prognosis and improve patient management. For example, using UHF MRI, it has been demonstrated that the rate of CL accumulation was higher in patients with RRMS who developed SPMS than in those who remained RRMS (3.6 lesions/year vs. 1.1 lesions/year, respectively), independently from the accumulation of WMLs (125). In MS, the use of 7T MRI showed a significantly higher number of focal MS lesions located in areas defined as NAWM on magnetisation-prepared rapid acquisition gradient-echo (MPRAGE) when compared with standard clinical 3T FLAIR (126). These abnormalities might have contributed to the pathological findings described below in the NAWM of MS. Also, exploiting the increased spatial resolution and enhanced contrast, UHF MRI can improve the detection and morphological characterisation of GM lesions (63). In addition, it improves the visualisation of the central vein sign (CVS) and the peripheral paramagnetic rim on susceptibility-weighted imaging (SWI) (127, 128). Imaging remyelination in MS lesions remains an unmet need. A straightforward approach to evaluate mechanisms of tissue repair and remyelination in chronic MS lesions has been recently proposed. Interestingly, the combination of a qualitative classification of lesions on MP2RAGE T1 maps with susceptibility-based 7T MRI seems to classify chronic lesions according to myelin content and to identify patients with a high risk for worse outcomes (129).

The use of UHF MRI may also help understand disease mechanisms. Indeed, in patients with NMOSD, a 7T MRI study on the periventricular venous density did not report alterations in the venous visibility on highly resolving T2^*^-weighted images, arguing against a widespread hypometabolism in NMOSD (130). Quantitative T1 relaxometry at 7 T was applied to assess structural alterations or damage in normal-appearing lesion-free periependymal regions of patients with NMOSD. In this study, a normal T1-relaxation time was found, which argues against a severe diffuse or “occult” brain damage even in AQP4-rich brain regions, thus supporting the findings from studies at 3T (131).

### Unmet Needs

Although MRI has gained an important role in the prognosis and better understanding of inflammatory demyelinating diseases, further research is warranted, particularly on MOGAD, for several reasons.

First, a significant unmet need of the research on MS is the discovery of mechanisms leading to disability worsening and disease progression. Beyond the development of novel imaging methods, the integration of multimodal data may facilitate the discovery of neuroprotective agents. For example, the rate of neurodegeneration may be assessed by integrating MRI with blood and CSF markers of neurodegeneration (e.g., neurofilament [NFL] levels) and ophthalmological imaging (i.e., OCT). Serum and CSF NFL light- and heavy-chain proteins have recently been proposed as reliable biomarkers of neuroaxonal damage in MS due to the correlation with MRI biomarkers of disability progression, such as brain and spinal cord atrophy (132). Nonetheless, the performance of NFL as a biomarker of neurodegeneration or neuroprotective treatment response in the progressive forms of MS is still uncertain and requires further research (133).

Second, although quantitative MRI techniques are technically challenging at the level of the spinal cord, they are able to provide valuable information on microstructural tissue damage. Multi-parametric imaging models are very important for the future, in order to look at the underlying driver of structural abnormalities. Indeed, sodium-MRI is a promising new metabolic imaging technique able to provide information on energy failure *in vivo*, and a protocol to detect sodium concentration in the cervical cord of MS patients has been recently developed, showing correlation with diffusion metrics (134). It would be interesting to combine structural and metabolic imaging measures in order to understand the mechanisms of damage and repair. This would be particularly useful in MOGAD, where a longitudinal study in a cohort of patients recruited soon after clinical onset would help understand the role of metabolic changes and myelin repair in contributing to the complete disappearance of lesions on conventional MRI.

Third, alongside neurodegeneration, remyelination is a possible mechanism of tissue repair as it may contribute to the shrinking of lesions in MS. Future longitudinal studies may assess whether a reduction in lesion size or lesion disappearance can be seen in MS and can be used as an outcome measure for repair treatments. Lesions may evolve differently in the two Ab-mediated disease diseases, and a complete resolution of T2 lesions occurs more frequently in MOGAD than AQP4-NMOSD and MS (135). Studying the evolution of lesions after an attack in the three CNS demyelinating diseases may guide treatment strategies, predict the disease course, and help to plan future clinical trials. Using imaging modalities sensitive to myelin changes (i.e., MTR, T1/T2 ratio), changes in myelin content of disappearing/shrinking lesions might be assessed in layers progressively further from the lesion core and inform about differential pathological mechanisms underlying MS and the two Ab-mediated diseases.

Moreover, when considering NMOSD, some previous studies did not separately analyse results by antibody type, therefore possibly including MOGAD patients which can also fulfil criteria for seronegative NMOSD. The distinction between AQP4 seropositive and seronegative NMOSD is further emphasised by recent prospective randomised clinical trials which included both groups but had an effect only on seropositive patients (136, 137). Thus, more homogeneous studies focusing on AQP4-seropositive cases are needed.

Finally, the role of new MRI biomarkers in the clinical management of inflammatory demyelinating diseases still needs to be established and the high cost of UHF MRI scanners makes it unlikely this type of imaging to be routinely and widely used in clinical practise in the near future.

## Conclusion

Conventional and non-conventional advanced MRI measures play an important prognostic role in inflammatory demyelinating diseases. The growing application of advanced imaging in MS, NMOSD, and MOGAD could improve the identification and validation of new pathological hallmarks in large clinical studies, leading to the development of novel diagnostic and therapeutic strategies.

## Author Contributions

RC designed the project and wrote the manuscript. AG designed the project and revised the manuscript. GS revised the manuscript. ND designed the project, supervised the project, and revised the manuscript. All authors contributed to the article and approved the submitted version.

## Conflict of Interest

The authors declare that the research was conducted in the absence of any commercial or financial relationships that could be construed as a potential conflict of interest.

## Publisher's Note

All claims expressed in this article are solely those of the authors and do not necessarily represent those of their affiliated organizations, or those of the publisher, the editors and the reviewers. Any product that may be evaluated in this article, or claim that may be made by its manufacturer, is not guaranteed or endorsed by the publisher.
